# Pharmacophore Elucidation and Molecular Docking Studies on 5-Phenyl-1-(3-pyridyl)-1*H*-1,2,4-triazole-3-carboxylic Acid Derivatives as COX-2 Inhibitors

**DOI:** 10.3797/scipharm.0912-19

**Published:** 2010-03-19

**Authors:** Marc Lindner, Wolfgang Sippl, Awwad A. Radwan

**Affiliations:** 1 Department of Pharmaceutical Chemistry, Martin-Luther-Universität Halle-Wittenberg, 06120 Halle, Germany; 2 Department of Pharmaceutical Organic Chemistry, Faculty of Pharmacy, Assiut University, Assiut-71527, Egypt; 3 Current address: Pharmaceutical Technology and Manufacturing Center, College of Pharmacy, King Saud University Al Ryiadh, Kingdom of Saudi Arabia, P. O. Box 2457, Riyadh, 11451, Kingdom of Saudi Arabia

**Keywords:** Catalyst, COX, GOLD, Docking, Pharmacophore

## Abstract

A set of 5-phenyl-1-(3-pyridyl)-1*H*-1,2,4-triazole-3-carboxylic acid derivatives (**16–32**) showing anti-inflammatory activity was analyzed using a three-dimensional qualitative structure-selectivity relationship (3D QSSR) method. The CatalystHipHop approach was used to generate a pharmacophore model for cyclooxygenase-2 (COX-2) inhibitors based on a training set of 15 active inhibitors (**1–15**). The degree of fitting of the test set compounds (**16–32**) to the generated hypothetical model revealed a qualitative measure of the more or less selective COX-2 inhibition of these compounds. The results indicate that most derivatives (**16**, **18**, **20–25**, and **30–32**) are able to effectively satisfy the proposed pharmacophore geometry using energy accessible conformers (E_conf_ < 20 kcal/mol). In addition, the triazole derivatives (**16–32**) were docked into COX-1 and COX-2 X-ray structures, using the program GOLD. Based on the docking results it is suggested that several of these novel triazole derivatives are active COX inhibitors with a significant preference for COX-2. In principle, this work presents an interesting, comprehensive approach to theoretically predict the mode of action of compounds that showed anti-inflammatory activity in an *in vivo* model.

## Introduction

In early 1990, isolation of cyclooxygenase isoforms and other findings had led to an initial hypothesis that cyclooxygenase-1 (COX-1) is responsible for the physiological function of prostaglandins (PGs), whereas COX-2 produces the “bad” PGs during inflammation processes. There are two central tenets of the hypothesis, firstly the PGs that mediate inflammation are produced solely via COX-2 and secondly PGs that are important in gastrointestinal and renal function are produced solely via COX-1. In the context of this hypothesis, the toxicity of NSAIDs in the gastrointestinal and renal systems is related to their lack of selectivity with respect to inhibition of COX-1 and COX-2 [1].

The major differences between the two iso-enzymes involve their dissimilarity in regulation and expression. COX-1 is almost detected in all cell types, although the level of expression varies among cell types. It is supposed to be the “Housekeeping Gene” which is essential for cytoprotection properties in stomach, haemostasis, platelets aggregation as well as renal sodium and water balance. In contrast, COX-2 is almost undetectable under normal physiological conditions, unless inflammatory mediators induce it [2]. The structural differences between COX-1 and COX-2 are due to two amino acid residues. Replacement of Ile523 in COX-1 by a relatively smaller Val in COX-2 is the most important difference between the two subtypes. This modification in the COX-2 enzyme allows access to an additional side pocket, which is a pre-requisite for COX-2 selectivity. A further important difference between the two enzymes is the exchange of His513 of COX-1 by the corresponding Arg499 in the COX-2 enzyme [3, 4]. Within the last few years several series of highly potent and selective COX-2 inhibitors have been reported [5–14].

Palomer et al. [15] described the construction of a pharmacophore using four known selective COX-2 inhibitors – celecoxib, rofecoxib, valdecoxib, and SC-558 – and considering the X-ray structure of COX-2 complexed with the selective inhibitor SC-558. The reported pharmacophore model shows a hydrogen bond acceptor, an aromatic ring (ring A) and an additional aromatic ring (ring B). Moreover, an excluded volume, which corresponds to Val523 was used for this structure-based pharmacophore [15].

5-Phenyl-1-(3-pyridyl)-1*H*-1,2,4-triazole-3-carboxylic acid derivatives (**16–32)** were recently synthesized in our group and tested in an animal model showing good anti-inflammatory activity [16]. We therefore considered it a worthy research endeavor to study the selective COX-2 inhibitory capability of these compounds by pharmacophore analysis and docking studies.

The Catalyst program [17] is generally used to analyze how ligands interact with a receptor by evaluating chemical features common to a set of active ligands (HipHop) [18] or by elucidating the correlation between activity and chemical binding features (HypoGen) [19]. One application of the Catalyst program is the generation of hypotheses that attempt to correlate the biological activity observed for a series of compounds to their chemical structures. The hypotheses are represented by chemical features that describe a series of compounds (e.g. hydrogen bonding acceptors (HBAs), positive and negative ionizable groups, etc). The hypotheses generated may be used to estimate the biological activity for potential drugs, allowing to rank potential synthetic priorities. In addition, the hypotheses generated may be used as three-dimensional queries to search databases of proprietary and/or commercially available compounds. In this study, we used the Catalyst program to establish a COX-2 pharmacophore by analyzing a variety of selective COX-2 inhibitors. The generated pharmacophore model was validated using a test set of triazole compounds (**16–32)** recently reported as anti-inflammatory agents. The COX-2 selectivity of these compounds was predicted using the Catalyst model. Complementary docking studies were carried out on the same set of triazole derivatives. The docking results suggested that these triazole derivatives are able to interact with the COX active site and are therefore suggested to be COX inhibitors with a clear preference for COX-2.

## Results and Discussion

### Common Feature-Based Pharmacophore Models

**Fig. 1.**
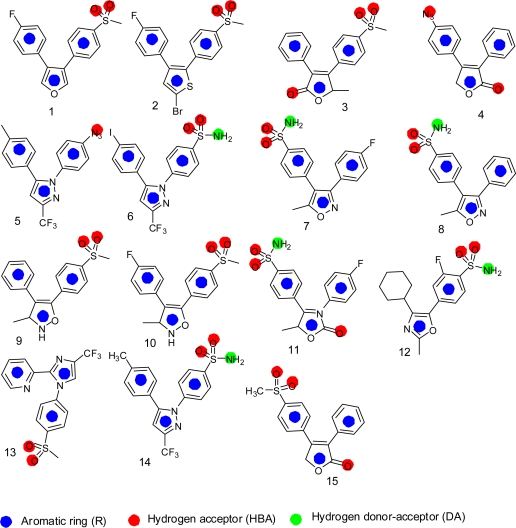
Structures of selective COX-2 inhibitors used in the Catalyst training set.

**Fig. 2.**
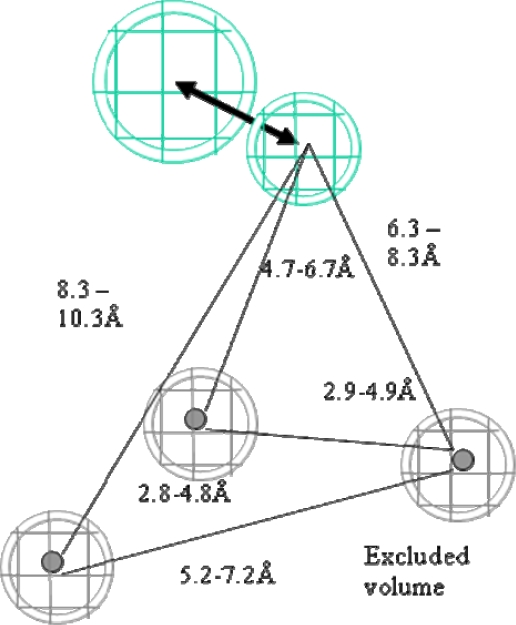
The top-ranked chemical feature-based pharmacophore model developed using the HipHop module in Catalyst. The pharmacophore includes two aromatic rings (gray) and one hydrogen bond acceptor feature (cyan). Distances are given in Angstrom.

**Fig. 3.**
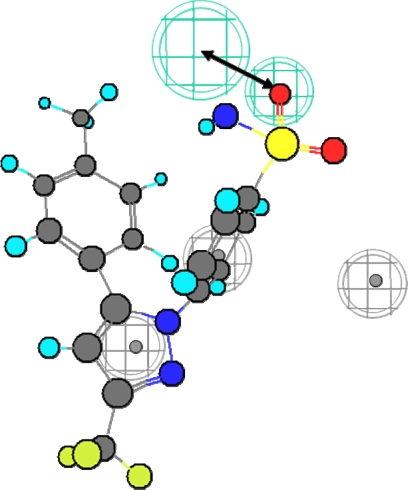
Compound 14 (celecoxib, training set) mapped to the HipHop pharmacophore.

Fig. 1 shows the structures of training set compounds (**1–15**) that are reported as selective COX-2 inhibitors [3, 20–30]. On the assumption that the most active compounds bind in a similar fashion at the enzyme’s active site, we employed the Catalyst/HipHop approach to evaluate the common features required for binding and the hypothetical geometries adopted by these ligands in their most active forms. Thus, these compounds were submitted for pharmacophore model generation based on common chemical features. Diverse conformation within 20 kcal/mol energy range were generated and submitted to the Catalyst/HipHop program. In the model generation methodology, the highest weighting was given to the most “selective” compound(s) in the training set (compounds **6**, **8**, **14**, **15**). Moreover, an excluded volume nearby the “selectivity filter” Val523 (which is known to be important for COX-1/2 selectivity) was set as sterically forbidden region in this structure-based pharmacophore. The crystallographic information was used to position an excluded volume in the pharmacophore accounting for the space limits imposed by this excluded volume [15]. The top-ranked chemical feature-based pharmacophore model identified in this study is shown in Fig. 2. This pharmacophore model contains three chemical features: two aromatic ring, R, (gray) and one hydrogen bond acceptor, HBA, (green). Fig. 3 shows as an example the alignment of the hypothesis model with compound **14** (celecoxib) of the training set.

### In silico prediction of selective COX-2 inhibiting activity and qualitative 3D-SSR analysis

**Fig. 4.**
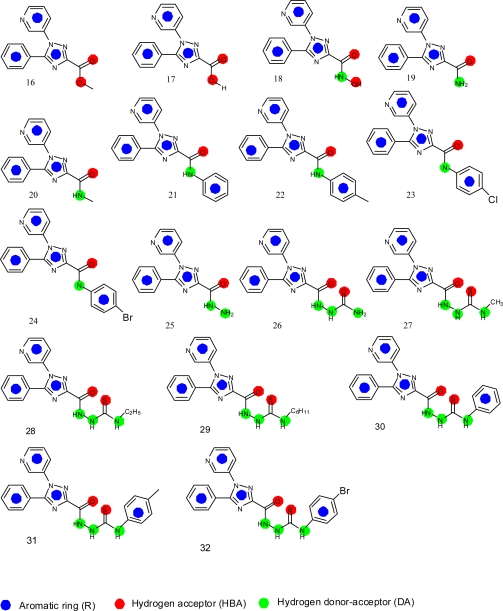
Structures of anti-inflammatory triazoles used in the Catalyst test set.

**Fig. 5.**
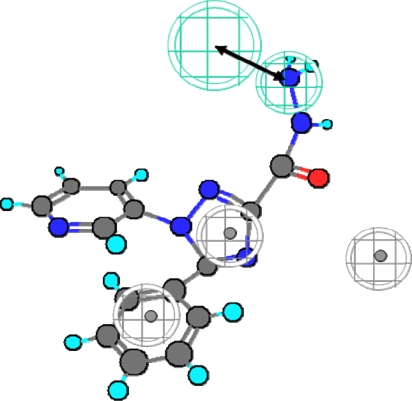
Compound **25** mapped to the HipHop pharmacophore.

**Fig. 6.**
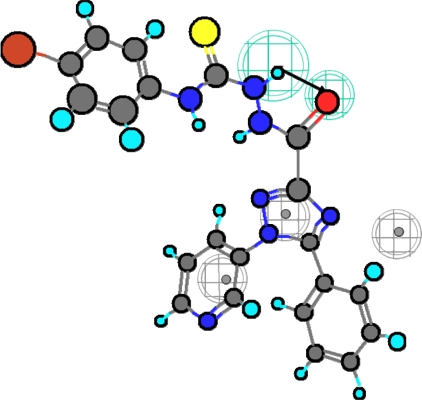
Compound **32** mapped to the HipHop pharmacophore.

This strategy intends to investigate the selective COX-2 inhibitory activity of a test set of 5-phenyl-1-(3-pyridyl)-1*H*-1,2,4-triazole-3-carboxylic acid derivatives (Fig. 4) [16]. As a readout, the HipHop program expresses the degree of mapping of a given compound to a generated hypothetical model in terms of fit values. The higher the fit value, the higher the expected activity against COX-2 enzyme. The Best fit method was used to map the chemical functions of each compound in the test set to the obtained hypothetical model and selects the most suitable alignment among its conformations. The fit values of the test set compounds obtained as well as their conformational energies are listed in Table 1. Fig. 5 and 6 show the alignment of the hypothesis model with compounds **25** and **32**, respectively, of the test set.

### Examination of the inhibitor binding site

A variety of COX-2-ligand crystal structures have been solved over the last years [3, 20, 21]. These X-ray structures provide important information about the relevant interaction possibilities at the COX-2 binding pocket. The COX-2 pocket is deeply buried within the protein at the end of a hydrophobic channel. The entrance of this channel is located nearby the membrane binding domain [22]. Structure–activity relationship data for first generation COX-2 inhibitors such as celecoxib and rofecoxib have shown that a SO_2_NH_2_, or a SO_2_Me substituent at the para-position of one of the aryl rings is a requirement for optimum COX-2 selectivity and potency. The X-ray structure 1CX2 with the co-crystallized celecoxib derivative SC-558 [23] is used to show the relevant protein-inhibitor interactions (Fig. 7a and 8a). The N-1 phenyl ring possessing the sulfonamide group is oriented in the vicinity of the COX-2 secondary pocket (nearby Ile517, Phe518). The sulfonamide accepts a hydrogen bond from the backbone NH of Phe518 (Fig. 7a). A second hydrogen bond is observed with His90. The para-bromophenyl-ring is oriented towards a hydrophobic pocket surrounded by the aromatic residues Trp387, Tyr385 and Phe518. The central pyrazole shows a hydrogen bond to Tyr355. The CF_3_-substituent is oriented toward the mouth of the COX-2 binding site, where it is located nearby Leu359 and Arg120 [24].

COX-1 has also been crystallized with a variety of inhibitors (Table 1) [22, 25–30]. As an example, Fig. 7b shows the COX-1 X-ray structure with the co-crystallized flurbiprofen. The carboxylate group is hydrogen bonded to Tyr355 and Arg120, while the aromatic rings are located in the hydrophobic area at the apex of the binding site. The terminal aromatic ring is stabilized by Leu352, Tyr385, and Trp387 (Fig. 7b).

### Docking results

In a first step we tested whether the docking program GOLD was able to reproduce the experimentally observed interaction mode of the 20 co-crystallized ligands (Table 2). Different docking settings and scoring functions were tested for the experimentally derived 20 COX-ligand complexes. The co-crystallized ligands can be classified based on their chemical structures: fatty acids, monocyclic inhibitors, bicyclic, or annealed tricyclic inhibitors. Docking with Goldscore and Chemscore showed comparable results and accuracy, whereas XScore [31] performed slightly worse (data not shown). Goldscore reproduced about 80–90% of the co-crystallized inhibitors correctly (rmsd within 2 Å, see Table 3). Most of the inhibitors were correctly predicted by GOLD, whereas the docking of the flexible fatty acids was less successful (see Tables 2 and 3). From the group of the inhibitors only diclofenac was not correctly docked. In a second step we tested whether using docking constraints (protein-ligand hydrogen bonds, hydrophobic constraints) could improve the docking accuracy. Using a combination of hydrophobic and hydrogen bond constraints extracted from the superimposed X-ray structures (Fig. 7a and 7b), a slight improvement in accuracy and correctness was observed (Tables 2 and 3).

The 17 synthesized compounds were subsequently docked into the COX-1 and COX-2 binding pocket using the same docking parameters as used for the validation set. The triazole derivatives show comparable interactions at COX-2 as the co-crystallized SC-558. As examples the docking solutions obtained for compounds **18** and **27** at COX-2 are shown in Fig. 8a and 8b in comparison with SC-558. The docked compounds fit well into the binding pocket and show hydrogen bonds with Tyr355, His90, Arg513, Gln192, Leu352, and the backbone NH of Ile517 and Phe518. The polar C3 substituent of the triazole ring fits into the COX-2 secondary pocket showing a similar interaction as the sulfonamide group of SC-558. One of the aromatic rings adopts the position of the central pyrazole ring of SC-558 whereas the second aromatic ring can be superimposed with the bromophenyl ring of SC-558.

In a next step we compared the binding mode obtained by docking with the Catalyst pharmacophore model. The same features as observed in the COX-2 pharmacophore were detected in the docking poses. The superimposition of the COX-2 pharmacophore with the COX-2/SC-558 complex is shown in Fig. 7c. The two hydrophobic features match the two aromatic rings of SC-558 used as docking constraints (Fig. 7a and 7c), whereas the hydrogen bond acceptor is located in close direction to Arg499. The excluded volume in the pharmacophore matches the position of Val523, which is known to be responsible for the COX-2 selectivity of the coxib derivatives.

In a next step we compared the binding mode obtained by docking with the Catalyst pharmacophore model. The same features as observed in the COX-2 pharmacophore were detected in the docking poses. The superimposition of the COX-2 pharmacophore with the COX-2/SC-558 complex is shown in Fig. 7c. The two hydrophobic features match the two aromatic rings of SC-558 used as docking constraints (Fig. 7a and 7c), whereas the hydrogen bond acceptor is located in close direction to Arg499. The excluded volume in the pharmacophore matches the position of Val523, which is known to be responsible for the COX-2 selectivity of the coxib derivatives.

Docking into the COX-1 binding pocket showed that the triazole derivatives show all a similar interaction mode. As examples, the obtained docking solutions for compounds **18** and **27** are shown in comparison with the co-crystallized inhibitor flurbiprofen in Fig. 9a, 9b. The central triazole ring adopts the same position as the central aromatic ring of flurbiprofen. The two aromatic rings of the ligands are oriented towards the hydrophobic pocket nearby Leu352. The polar substituent attached to the triazole is hydrogen bonded to Tyr355 and Arg120 as the carboxylate of the COX-1 inhibitors. Due to the fact that no tricyclic inhibitor has been co-crystallized with COX-1 yet, the interaction mode of the synthesized derivatives is more speculative. Comparison of docking scores that we obtained for the co-crystallized inhibitors, the training- and test set molecules yielded a preference of the triazole derivatives for COX-2. Therefore, based on our docking results it is suggested that the synthesized triazole derivatives are active COX inhibitors with a clear preference for COX-2.

We had no access to COX in vitro assay data and thus it was not able to carry out a quantitative structure activity relationship. However, several compounds of the training set were recently tested in animal models for their anti-inflammatory activity and their gastric ulceration effects [16]. Compounds **16** and **25** showed no effect in the ulceration test, while compounds **24** and **32** caused gastrointestinal lesions in a dose-dependent manner. It is known that gastric ulceration effects of COX inhibitors are mainly the results of blocking the COX-1 mediated pathway. The derived docking scores (Table 4) for the COX-1 inhibitors are in qualitative agreement with the effects measured in the gastric ulceration model. Compound **24** was the top-scored COX-1 inhibitor among all docked compounds. On the other hand, compound **25**, the most active one in the anti-inflammatory model, was found to be the top-scored inhibitor for COX-2.

## Methods

### Common Feature-Based Pharmacophore Models. Selective COX-2 Inhibitors

The study was performed using the Catalyst 4.7 software [17] on a 195 MHz MIPS R10K Octane (IRIX64). All compounds were constructed using the 2D/3D sketcher of the Catalyst software. By use of the conformational poling approach [32], a conformational model was generated for each compound within a 20 kcal/mol range above the calculated energy minimum (BEST search method in Catalyst). The numbers of calculated conformations range from 11 to 106 for the different compounds.

Feature-based 3D pharmacophore alignments of COX-2 inhibitors were performed using the Catalyst/HipHop program [33, 34]. This was carried out in a three-step procedure: [35] (a) a conformational model for each molecule in the training set of 15 selective COX-2 inhibitors was generated; (b) each conformer was examined for the presence of chemical features; (c) a three-dimensional configuration of chemical features common to the input molecules was determined. Catalyst provides a dictionary of chemical features found to be important in drug-enzyme/receptor interactions. These are hydrogen bond donors, hydrogen bond acceptors, hydrophobic groups, and positive and negative ionizable groups. For the pharmacophore modeling, common features selected for the run were aromatic ring (R) and hydrogen bond acceptor (HBA) functions.

### In silico prediction of COX-2 inhibiting activity and qualitative 3D-SSR analysis

A set of 5-phenyl-1-(3-pyridyl)-1*H*-1,2,4-triazole-3-carboxylic acid derivatives (**16–32**) that have been reported as anti-inflammatory agents, were allowed to fit to the generated hypothetical model of selective COX-2 inhibitors. The crystallographic information was used to position an excluded volume in the pharmacophore accounting for the space limits imposed by this excluded volume [15]. Best conformational analysis was performed for each compound using a threshold of 250 conformers per molecule and a maximum value of 20 kcal/mol for conformer energy. Subsequently, the conformers of each synthesized compound were allowed to fit to the generated hypothetical model utilizing the best fit method. The obtained fit value for each molecule is a measure of how many and how well its functional features fit to the features of the pharmacophore.

### Docking study

All calculations were performed on a Pentium IV 1.8 GHz based Linux cluster. The molecular structures of the ligands were generated using Sybyl 7.1 [36]. The ligand structures were energy minimized using the MMFF94s force field and the conjugate gradient method, until the default derivative convergence criterion of 0.05 kcal/(mol Å) was reached. All compounds were generated in the protonation state assuming physiological conditions.

The coordinates of 13 Cyclooxygenase-1 (COX-1) and 7 Cyclooxygenase-2 (COX-2) X-ray structures were taken from the Protein Data Bank [37]. COX-1 and COX-2 are monomers in solution, and therefore only one chain from the available X-ray structures was considered for docking. To validate the GOLD docking we used the 20 X-ray structures as validation set (Table 1). The 20 co-crystallized ligands were docked into their original protein structures after removing all co-crystallized ligands and solvent molecules. For the docking of the developed inhibitors we selected the X-ray structures of 1EQH and 1CX2 as representatives for COX-1 and COX-2, respectively.

Docking of all ligands was carried out using the program GOLD 3.0 [38] (Cambridge Crystallographic Data Centre, CCDC). Docking was performed with default settings to obtain a population of possible conformations and orientations for the inhibitors at the binding site. A 10 Å sphere around the centre of the binding pocket was defined as binding pocket for the docking. All torsion angles in each compound were allowed to rotate freely. Goldscore, Chemscore and, Xscore [31] were tested as fitness functions. The resulting solutions were clustered on the basis of the heavy atom rmsd values (1 Å). For each molecule, 10 individual docking runs were performed. The docking poses for each ligand were stored in a MOE database and further processed using in-house MOE-SVL-scripts (Chemical Computing Group, CCG) [39].

## Conclusions

In this work we have developed a pharmacophore hypothesis and carried out docking studies on 5-phenyl-1-(3-pyridyl)-1*H*-1,2,4-triazole-3-carboxylic acid derivatives as COX-2 inhibitors. These studies resulted in a reliable COX-2 pharmacophore model and allowed us to shed light on the binding features of these derivatives to COX-1 and COX-2 which showed a clear preference for COX-2. The derived pharmacophore model were found to be in agreement with the structural features at the COX-2 binding site. These findings could be exploited for future ligand design in order to obtain novel triazole derivatives as selective COX-2 inhibitor.

## Figures and Tables

**Fig. 7a.**
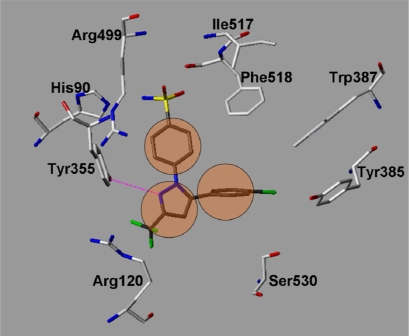
COX-2 docking constraints: For comparison the co-crystallized celecoxib derivative SC-558 (from 1CX2.pdb) is shown (coloured dark gray). The orange circles indicate the applied hydrophobic constraints whereas the hydrogen bond constraint is shown in magenta.

**Fig. 7b.**
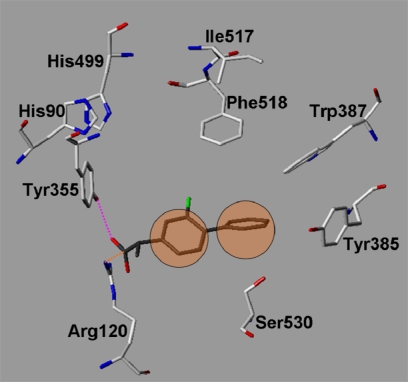
COX-1 docking constraints: For comparison the co-crystallized inhibitor Flurbiprofen (from 1CQE.pdb) is shown (coloured dark gray). The orange circles indicate the applied hydrophobic constraints whereas the hydrogen bond constraints are indicated by dashed lines.

**Fig. 7c.**
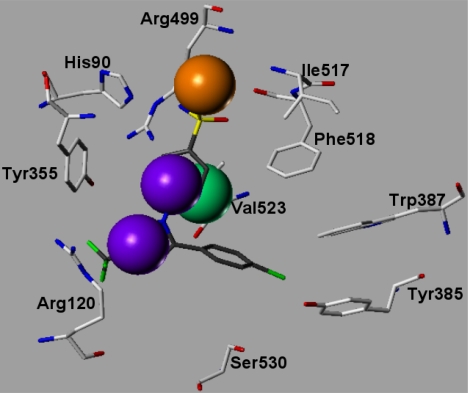
Comparison of the COX-2/SC-558 complex obtained from the crystal structure and the Catalyst COX-2 pharmacophore. The violet spheres indicate the two aromatic features whereas the hydrogen bond acceptor is shown in orange. The excluded volume (green) of the pharmacophore coincides with Val523 of COX-2.

**Fig. 8a.**
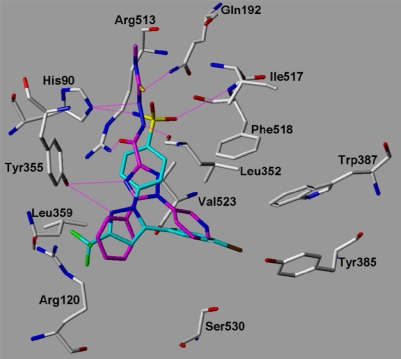
COX-2: superimposition of the co-crystallized Celecoxib derivative SC-558 (from 1CX2.pdb, coloured cyan) and the docked compound **27** (coloured magenta). Hydrogen bonds are displayed in magenta.

**Fig. 8b.**
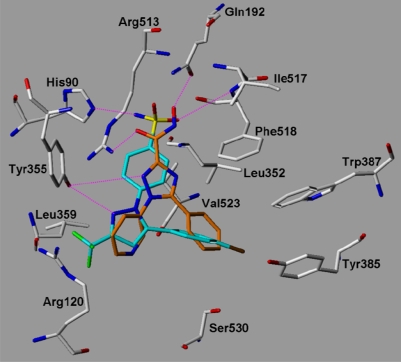
COX-2: superimposition of the co-crystallized celecoxib derivative SC-558 (from 1CX2.pdb, coloured cyan) and the docked compound **18** (coloured orange). Hydrogen bonds are displayed in magenta.

**Fig. 9a.**
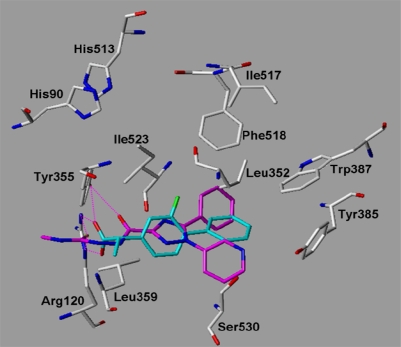
COX-1: superimposition of the co-crystallized flurbiprofen (from 1EQH.pdb, coloured cyan) and the docked compound **27** (coloured magenta). Hydrogen bonds are displayed in magenta.

**Fig. 9b.**
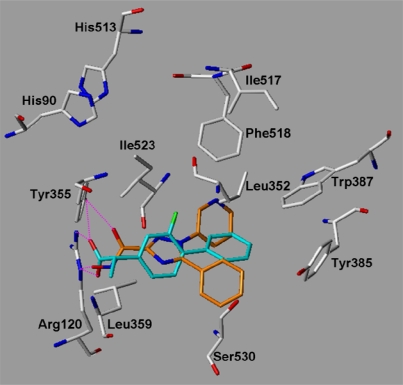
COX-1: superimposition of the co-crystallized flurbiprofen (from 1EQH.pdb, coloured cyan) and the docked compound **18** (coloured orange). Hydrogen bonds are displayed in magenta.

**Tab. 1.** Fit values of the investigated compounds **16–32** (test set) to the hypothetical model.

**Compound number**	**No. of conformers**	**Fit value**	**Energy of best fitting conformer**
16	18	2.88	4.10
17	23	2.12	0.06
18	21	2.72	0.09
19	106	2.57	0.001
20	83	2.87	0.00
21	26	2.87	4.09
22	15	2.88	4.01
23	49	2.90	1.06
24	40	2.88	0.20
25	11	2.90	0.10
26	74	2.61	10.47
27	37	2.56	3.09
28	44	2.58	4.10
29	52	2.63	3.27
30	11	2.87	4.70
31	36	2.89	5.08
32	24	2.90	8.02

**Tab. 2.** COX protein-ligand X-ray structures used for the docking study. The rmsd values (heavy atoms) between experimentally observed and docked ligand structure (top rank) are indicated.

**Pdb code**	**Co-crystallized ligand**	**Subtype**	**Ref.**	**RMSD (top rank)**
1CQE	Flurbiprofen	COX1	[22]	0.43
1Q4G	2-(1,1′-Biphenyl-4-yl)propanoic acid	COX1	[27]	0.32
1DIY	Arachadonic acid	COX1	[26]	3.65
1EQG	Ibuprofen	COX1	[30]	0.23
1EQH	Flurbiprofen	COX1	[30]	0.23
1FE2	Eicosa-8,11,14-trienoic acid	COX1	[29]	11.37
1HT5	Flurbiprofen methylester	COX1	[30]	1.03
1HT8	(3-Chloro-4-propoxyphenyl)acetic acid	COX1	[30]	1.29
1IGX	5,8,11,14,17-Eicosapentaenoic acid	COX1	[25]	2.49
1IGZ	Linoleic acid	COX1	[25]	1.72
1PGE	Iodosuprofen	COX1	[28]	0.55
1PGF	Iodoindomethacin	COX1	[28]	0.76
1PGG	Iodoindomethacin	COX1	[28]	1.04
1CVU	Arachadonic acid	COX2	[25]	1.22
1CX2	SC-558	COX2	[3]	1.01
1DDX	Prostaglandine G2	COX2	[21]	4.63
1PXX	Diclofenac	COX2	[20]	5.83
3PGH	Flurbiprofen	COX2	[3]	0.40
4COX	Indomethacin	COX2	[3]	0,78
6COX	SC-558	COX2	[3]	0.40

**Tab. 3.**
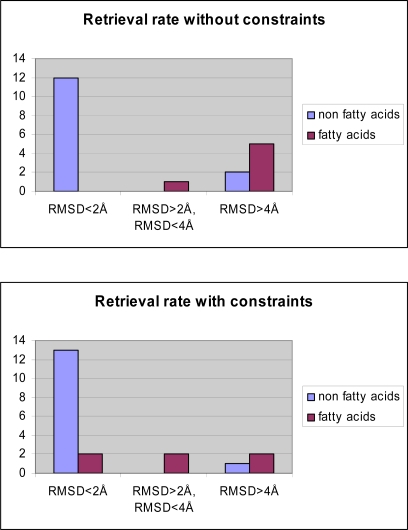
GOLD/GoldScore docking results for the 20 co-crystallized ligands. Twelve of the co-crystallized inhibitors can be reproduced within 2 Å (retrieval rate: 86%) and thirteen by using constraints (retrieval rate: 92%).

**Tab. 4.** Calculated docking scores for COX-1 and COX-2.

**Inhibitor**	**GoldScore COX-1**	**GoldScore COX-2**
16	44.7	49.1
24	58.1	51.8
25	44.6	61.7
32	53.5	45.3
